# Recollections of Hospital Life

**Published:** 1887-12-10

**Authors:** 


### Recollections of Hospital Life.

By an Ex-Patient.

The following "Recollections" have been sent to us by the physician who was in charge of the case. The patient was a well-known and highly-respected reporter and contributor to the *South London Press*. He writes:

Until a certain month in a certain year all my notions of hospital treatment were derived from what I had heard at meetings of anti-vivisectionists. Hence the medical student was to me a kind of fiend in human form, taking especial delight in torturing animals, perhaps even a fellow-creature, and I had a vague impression that physicians were capable of allowing their own grandmothers to be cut up if they could thereby advance science, or, in other words, add something to their professional knowledge. It was therefore with some alarm that I listened to my doctor when he suggested that I should go into the hospital on the ground that in one of these institutions I should derive more real good in one month than he could possibly do me in three months. "Don't you think, doctor," I ventured to ask, "they are sometimes tempted to make a case out of you?" He smiled, but assured me that in this enlightened nineteenth century it was the ambition of medical gentlemen to effect as many cures as possible for the credit of the hospital to which they might be attached. Somewhat reassured, although not quite convinced, I followed the advice of my doctor, and sought admission to an hospital. I shall never forget, however, the feeling with which I awaited the appearance of the resident medical officer, when, after a tedious journey by cab, I ultimately found myself with some fifty or sixty other victims in a large waiting-room. And when at length he did come, I could not but think what little chance with him any one would have who might not possess the necessary qualification for admission—some real complaint needing hospital treatment. He was sharpness itself, although every inch a gentleman; and with a rapidity almost astonishing he sifted out the genuine cases. I had the felicity of retiring behind a screen, where I was subjected to an examination of a crucial character. He did not, of course, condescend to gratify my curiosity by stating what he thought about my case, but I had no difficulty in drawing a correct inference, when an obliging attendant tapped me on the shoulder, and remarked, "You're admitted. Come this way." Even at this stage I was struck with the appliances provided for the comfort of the sick and maimed. I was placed in a wheeled chair, and in a comparatively short time I had in this easy way traversed a long corridor, and (by means of a lift) had ascended to one of the upper wards of the hospital. And these wards! Shall I ever forget what pictures of cleanliness and comfort they appeared to me? "Can this be," I thought, "the place I have so much dreaded?" I cannot say whether I was lucky in being sent to the particular ward in which I found myself, but this I will say—that if the Sisters in the other wards were like our Sister; if the staff-nurses in the other wards were like our staff-nurses; and if the "lady" nurses and "Nightingale" nurses were like our "ladies" and our "Nightingales," then the patients of this hospital were indeed highly favoured. There was discipline, it is true, but it was so mingled with genuine interest and regard for the patients that it was scarcely felt.

On first entering the ward I was struck by the evidences I saw in every direction of the order and cleanliness to which so much importance is attached in these institutions. I had only been in the ward a few minutes when I had a practical proof of the latter, inasmuch as I was handed over to Old Peter the bathman, with an intimation that I was to submit to the ordeal of a warm bath. Old Peter—I call him such for want of his real name—was the kindest-hearted man I have ever known. In his hands the most feeble patient was perfectly safe. He had grown old in the occupation of bathing poor wrecks of humanity, and his skill was only equalled by his gentleness and consideration for the patients. Few things I have ever enjoyed more than the baths I had whilst in hospital; and who can wonder, when Old Peter would come to my bedside, envelop me in a blanket, wheel me to the bath, attend most carefully to me whilst in it, and then, again enveloped me in the blanket, deposit me in bed and see me comfortably tucked in before wishing me "good morning"?

The greatest trial to me at first was the monotonous regularity of the meals. To be expected not to get hungry before six o'clock in the morning was something I could not understand, and I must confess that I used to listen with feverish anxiety for the clock to strike six, for with unvarying punctuality our kind night nurse would make her appearance with the "mugs" and the "plates"—the forerunners of the breakfast for the thirty patients in our ward. Until this time, talking was not presumed to have taken place, and hence there was always a strong reaction, and the thirty patients would often be nearly all talking at once. All the patients are looked upon as so many "numbers," and in time I came to regard myself as "Number Twenty," and felt quite surprised if, by an accident, my name was mentioned. At breakfast time, therefore, it was rather amusing to hear inquiries such as "Number Sixteen, how do you feel this morning?" Sixteen replies that he has spent a bad night, and he in his turn is solicitous as to the condition of "Number Twenty-five"; and so these kind inquiries would go on until every other patient knew exactly how every other patient had spent the night, and was at that particular time. Talk about a number of old women gossiping, it is nothing to the talking between thirty grown-up men when they find themselves caged up in the ward of an hospital!

After breakfast it was generally a busy time. Such of the patients as could walk would retire to the lavatory to perform their ablutions, but the majority of the patients needed to be indulged with basins of tepid water at their lockers, and in some cases the nurses would help the patients to make themselves smart. Then there was bed-making. Not the simple bed-making of home, but the complicated task of making a bed so as not to put a patient to the least inconvenience—something very necessary in bad cases where the slightest movement is often torture to the patient. When convalescent I was permitted to make my own bed, and I have a vivid recollection of standing a few minutes wondering where the bolster-case was. I had forgotten that the sheet was required to be artfully brought over the bolster, and then artistically tucked in.

At eight o'clock, no matter what was being done, prayers were read by the Sister. We used to call her "our Sister," and I fancy there was not one of us but would have considered it a great punishment if we had been handed over to any other Sister. When she retired to rest I never could make out, for whenever I have accidentally opened my eyes, I have generally seen her flitting noiselessly about the ward seeing that this or that had not been neglected. Truly a noble work it is in which these Sisters and nurses are engaged! They little know the gratitude of the patients upon whom they attend—I speak now of patients generally, and not of those exceptional persons upon whom all kindness is thrown away. I have mixed freely with the patients in the ward where I had to remain, I am sorry to say, some months; and I was often struck by the kindly feeling—a feeling almost amounting to affection—entertained towards the Sisters and nurses. Personally, I cannot but regard the hospital, of which I am writing, as a second home—a place where I received nothing but kindness and where I made many friends; a place, too, where many previous impressions of men and things received some rude shocks. Of course there are many routine matters in connexion with hospital life which must be passed over, but in thus dismissing them it must not be assumed that they are unimportant. I could say a good deal about the Nightingale nurses, but as I am rather susceptible to the influence of the fair sex, I fancy it will be judicious not to do so, merely remarking that I think their costume most coquettish and becoming, and that I have a strong suspicion some of them are quite aware of this circumstance. As soon as the ward had become a perfect picture of order, comfort, and cleanliness, the "clinicals" used to visit the patients. So far as my experience went, I found them most gentlemanly and considerate. They would listen with the greatest attention to the patients whilst the latter entered into details concerning their complaints—and terribly long yarns some of the patients indulged in. When these young gentlemen had duly dotted down any fresh symptoms in the cases, the house surgeons would come round and devote a good deal of attention to each case, whilst the resident medical officer was perfectly ubiquitous, and seemed intuitively to know just those cases more particularly requiring his attention. About this time the patients would be served with lunch, and then there would be a lull until dinner time. It was at the dinner table of this particular hospital that I became celebrated as an expeditious peeler of potatoes. Sometimes, I must confess, economy was sacrificed to velocity, and hence many a potato by the time it had passed through my hands had become small and beautifully less; but I had always this consolation, that I never left a particle of the skin on. I will do the hospital authorities justice by recording the fact that the mutton we had for dinner was of the best quality. I am not quite certain what took place after dinner, but I have a general impression that most of the patients vanished under the sheets, and resigned themselves to Morpheus. At half-past four we had tea, subsequently prayers, then supper; and at eight o'clock the sorest trial of all—"No more talking!" Some of the patients of a Jesuitical turn of mind argued that this did not include "whispering," and hence conversations of the most edifying character have been carried on without the officials deriving that benefit from them which otherwise they might have done. During the whole of the day the patients were in danger of being killed with kindness. Several times a day there was medicine —I didn't mind this, as my medicine was pleasant to the taste, but to some these periodical doses must have been far from agreeable; and at this moment I cannot recollect how many times a day the beds were made comfortable for the patients. I don't believe there is a harder-worked person than a hospital nurse; and yet there are scores of ladies performing the duties of the position cheerfully. I can conceive of no other position requiring such self-command and such self-sacrifice.

After the patients had been made comfortable for the night, it was usual for the house-surgeons to visit the ward and inquire into each case. And now about visiting days. These were of two kinds. Twice or more a week the physicians visited the hospital. On such occasions there were great efforts to paint the lily; in other words, to make the ward look more perfect than it was normally—an almost impossible task. To a new patient the arrival of the physician, accompanied by some twenty or thirty students, was generally a source of uneasiness, particularly if he imagined that an operation might have to be performed. I felt certain that I was doomed to be operated upon, and that, at any rate, was my feeling. At such visits it is a significant sign when the screen is placed round a patient, and physician and student surround his bed. When the screen was placed round my bed, I called to mind all the horrible stories I had heard from anti-vivisectionists; but I soon became reconciled to my fate, and bore the operation with, I hope, as good a grace as possible —at least with as much patience as I could muster. I am bound to state that from the physician to the youngest student present I received the most considerate attention, and I fully believe that the operation was performed with a minimum of pain to myself. During the whole of the time I was in hospital I saw nothing in the conduct of the students to justify some of the vulgar prejudices entertained concerning them. On the contrary, the majority of them seemed only too anxious to derive that practical insight into the medical profession which hospital practise undoubtedly affords. Into the various operations I saw performed I have no business to enter; so I will pass on to the other class of visitors—the friends of the patients. The days when such visitors were permitted to come were anxiously expected by the patients. None but those who have spent a month or two in an hospital can understand the feeling with which such visiting days are anticipated, and the regret with which patients see their friends leave the ward. One hour and a half was the time allowed, and the patients had generally so much to communicate, and the visitors also so much to communicate, that the time seemed to fly, and often conversations had to be broken off just at those critical points where in tales one reads "To be continued in our next." Many affecting scenes are associated with these visiting days, for in many cases friends leave the ward with the knowledge that in all probability the patient they have visited will not be in his place at the next visiting day. During the months I was an inmate I saw many new faces and many distressing sights which I shall never forget. I cannot conceive of anything more humbling than to see men of splendid physique confined to their beds. In most of such cases the cause is aneurism. Many wonderful cures I saw effected. Some of the patients told me that they all thought I should never leave the ward alive. I was under the same impression. There were several whom I gave up, but afterwards had the satisfaction of seeing walk out of the ward quite recovered. After this lapse of time, I care not to recall the many distressing scenes I witnessed: I prefer to speak of the friendships formed in hospital, and of the way in which the wealthy might render life in such a place less monotonous. In an hospital ward social distinctions are forgotten, and patients freely tell one another matters which, out of the hospital, would never be mentioned. I am sorry to say that in nearly every case there was a tale of suffering and privation which never could have been guessed from the cheerfulness of the patients. What is the inference I wish to be drawn from this? It is that in our hospitals nearly every bed contains a patient whose anxieties would be relieved, and who would be able to endure more bravely his sufferings, if some kind friend would unobtrusively discover and relieve the hardships which his presence in hospital may have entailed upon wife and children, or father and mother, as the case may be. In my own case it was an unspeakable satisfaction to know that kind friends had not forgotten to provide for those at home. And then to relieve the monotony inseparable from life in an hospital I know of no more acceptable boons than good books. My friends kept me well supplied, and it was gratifying to me to find constant applications for them. I know I am touching on dangerous ground when I venture to indicate what books should be given or lent, and that being so, I will content myself by saying that among the best are undoubtedly standard novels. As a rule, works of a serious character are always to be obtained in most hospitals. In concluding these rambling recollections, I shall feel myself amply repaid if I succeed in inducing any reader to take a greater interest in the poor creatures to be found in our hospitals.

